# The effects of progressive muscle relaxation on sleep quality in adults: a systematic review and meta-analysis of randomized controlled trials

**DOI:** 10.3389/fpubh.2026.1906525

**Published:** 2026-08-06

**Authors:** Kehan Li, Shiwei Chen, Yixuan Liu, Nanding Yu

**Affiliations:** 1 Department of Sports Studies, Faculty of Educational Studies, Universiti Putra Malaysia, Serdang, Malaysia; 2 School of Health Sciences, Universiti Sains Malaysia, Kubang Kerian, Malaysia; 3 Center for Healthy Ageing and Wellness (HCARE), Faculty of Health Sciences, Universiti Kebangsaan Malaysia, Kuala Lumpur, Malaysia

**Keywords:** adult, meta-analysis, Pittsburgh Sleep Quality Index, progressive muscle relaxation, sleep

## Abstract

**Background:**

With the increasing number of people suffering from insomnia, sleep has evolved into a global public health issue. Compared to the side effects and instability of drug treatments, people are constantly seeking non-pharmacological treatments to improve sleep quality. Progressive muscle relaxation (PMR) has gradually come into focus. Because existing evidence remains inconsistent, this study conducted a systematic review and meta-analysis.

**Methods:**

As of May 2026, studies related to progressive muscle relaxation and sleep quality were identified through searches of five databases, including CINAHL, Embase, PubMed, Scopus, and Web of Science. Sleep quality outcomes were synthesized using a random-effects meta-analysis. analyses were conducted according to participant type (cancer patients and non-cancer patients) and age group (Age < 55 years and Age ≥55 years). Meta-regression was used to examine whether the number of progressive muscle relaxation sessions was associated with sleep quality. Sensitivity analysis and publication bias assessment were performed.

**Results:**

Fourteen studies involving 957 participants were included in the final analysis. The pooled findings indicated that progressive muscle relaxation improved sleep quality (Hedges *g* = −1.24, 95% CI −1.71–−0.77, *p* < 0.001, *I*^2^ = 85.5%). Improvements were observed in both cancer patients (Hedges *g* = −1.17, 95% CI −2.24–−0.09, *I*^2^ = 86.8%) and non-cancer patients (Hedges *g* = −1.29, 95% CI −1.94–−0.65, *I*^2^ = 86.5%), with a larger effect observed among non-cancer patients. When participants were grouped by age, a significant benefit was found in adults younger than 55 years (Hedges *g* = −1.40, 95% CI −2.12–−0.69, *I*^2^ = 89.4%), whereas no significant improvement was detected among adults aged 55 years and older (Hedges *g* = −1.17, 95% CI −2.67–0.32, *I*^2^ = 77.5%). Meta-regression did not identify a significant association between the number of progressive muscle relaxation sessions and sleep quality (*p* = 0.2106).

**Conclusions:**

The available evidence suggests that progressive muscle relaxation improves subjective sleep quality in adults. Beneficial effects were observed in both cancer and non-cancer populations, whereas the effects among older adults remain uncertain. Sleep quality improvements also do not appear to be related to the number of progressive muscle relaxation sessions.

**Systematic review registration:**

https://www.crd.york.ac.uk/PROSPERO/view/CRD420251267826, Identifier: CRD420251267826.

## 1 Introduction

Sleep is defined as a complex natural behavior and an important part of a healthy life (1). Sleep affects people's physical and mental health to varying degrees (2). Studies have shown that insufficient sleep may increase the risk of chronic diseases such as cardiovascular and cerebrovascular diseases, diabetes, hypertension and obesity (3, 4). Both long-term and short-term sleep problems may lead to cognitive risks such as cognitive impairment or memory decline. This is especially true in older adults (5, 6). In contrast, people with poor sleep quality are more likely to experience depression, anxiety and aggressive behavior (7, 8). According to epidemiological statistics, approximately 30%−35% of adults worldwide suffer from varying degrees of sleep problems, and this proportion continues to increase with age (9, 10). As sleep problems have gradually evolved into a global public health issue, non-pharmacological interventions have attracted increasing attention because of the potential adverse effects associated with pharmacological treatments (11).

Progressive muscle relaxation (PMR), originally proposed by Jacobson in 1929 and further developed in 1938, is one of the most widely used non-pharmacological interventions (12, 13). It relieves muscle tension and relaxes nerves by coordinating regular breathing with the contraction and relaxation of muscles throughout the body (14). These physiological and psychological responses are thought to reduce hyperarousal, alleviate anxiety, decrease sympathetic nervous system activity, and enhance parasympathetic activity, thereby contributing to improvements in sleep quality. (14–16). Initially, people used the reciprocal inhibition mechanism of progressive relaxation to treat depression and anxiety (15). Later, it was discovered that this therapy, which relaxes the whole body muscles and relieves nervous tension, can reduce fatigue and improve sleep quality (16). Because PMR is simple, inexpensive, not restricted by location, and associated with few adverse effects, it has received increasing attention as a complementary therapy for sleep problems (17).

However, findings on the effect of PMR on sleep quality remain inconsistent. Some studies reported that PMR did not improve overall sleep quality, although improvements were observed in some subscales (18). Although previous systematic reviews have suggested that PMR may improve sleep quality in patients with chronic diseases, they did not examine whether the effects of PMR varied according to participant characteristics, age, or intervention frequency (19). These inconsistencies may be attributable to differences in participant characteristics, underlying diseases, age, intervention protocols, and outcome assessment methods. Sleep disturbances are particularly common among individuals with chronic diseases, especially patients with cancer, highlighting the potential value of PMR as a complementary intervention in clinical settings (20–22).

Considering these inconsistent findings, a comprehensive synthesis of the available evidence is warranted. Although previous studies have investigated the relationship between PMR and sleep, important uncertainties remain regarding whether intervention effects differ across participant characteristics and whether intervention frequency influences treatment outcomes. Therefore, this study used the Pittsburgh Sleep Quality Index (PSQI) as the primary outcome measure and conducted a systematic review and meta-analysis to examine the effect of PMR on subjective sleep quality. Subgroup analyses based on participant type and age, together with meta-regression analysis, were performed to explore potential sources of heterogeneity. This study aims to provide a comprehensive synthesis of the current evidence to inform clinical practice and future research regarding the use of PMR for improving subjective sleep quality.

## 2 Methods

In accordance with the requirements of the PRISMA guidelines, to ensure the stability and rigor of the study, this systematic review and meta-analysis were pre-registered in the PROSPERO database (registration number: CRD420251267826) (23).

### 2.1 Search strategies and selection

Literature searches were carried out in five databases: CINAHL, Embase, PubMed, Scopus, and Web of Science. No starting date restriction was applied, and the final search was completed in May 2026. We used Boolean equations to search for keywords related to Progressive Muscle Relaxation (progressive muscle relaxation, muscle relaxation therapy, Jacobson relaxation, Jacobson technique, PMR), Sleep (sleep, sleep wake disorders, sleep quality, insomnia, sleep disturbance, sleep disorder, sleep problem), and Randomized Controlled (Randomized Controlled Trial, Controlled Clinical Trial, randomized, randomly, trial), using OR or AND connections (See Supplementary Appendix for details). Two researchers (KH L and YX L) independently screened the literature using a blinded method. If any discrepancies arose, they were discussed and resolved with a third researcher (SW C).

### 2.2 Inclusion and exclusion criteria

#### 2.2.1 Inclusion criteria

P (study population): Participants were adults >18 years of age, regardless of sex or disease status.

I (intervention group): PMR was used as the intervention, including any type (Jakobson PMR, modified PMR, and audio-integrated PMR), frequency, and duration.

C (comparison group): No type of PMR was performed. This included no intervention, control group, sleep education, health education, and routine care.

O (outcome): PSQI was used as the assessment indicator of sleep quality. Only studies using the PSQI were included to ensure methodological consistency across studies. Other validated sleep assessment tools may evaluate different sleep constructs (e.g., insomnia severity or daytime sleepiness) or objective sleep parameters, and therefore were not combined in the present meta-analysis.

S (study design): Only randomized controlled trials (RCTs) were included.

#### 2.2.2 Exclusion criteria

P (study population): Participants under 18 years of age or animal studies.

I (intervention group): Experiments that did not use PMR as an intervention or interventions combined with other therapies.

C (comparison group): Not applicable.

O (outcome): PSQI outcome was not reported.

S (study design): Non-randomized controlled trial, conference abstract, commentary article, article with incomplete data, unpublished study.

### 2.3 Data extraction and coding

After excluding duplicate studies, two researchers (KH L and YX L) conducted an initial screening of titles and abstracts independently and cross-checked. Studies that did not meet the pre-defined inclusion and exclusion criteria were excluded. The articles after the initial screening were read in full to determine those eligible for inclusion. If the two researchers disagreed, they were resolved through discussion with a third researcher (SW C). Extracted information included authors, publication year, country, number of participants, gender, age, participant origin, intervention method, control method, intervention duration, and outcome measure PSQI. In this study, the outcome measure was the total PSQI score. For studies that did not report specific numerical values in the text, we obtained them by emailing the corresponding authors.

### 2.4 Quality assessment

Following the recommendations of the Cochrane Handbook, the methodological quality of all eligible studies was evaluated. The Risk of Bias Assessment Tool version 2.0 (ROB2) was used for the quality assessment (24). Five domains were examined in ROB2, including the randomization process, deviations from intended interventions, missing outcome data, outcome measurement, and selective reporting. Each aspect was rated as “low risk,” “some issues exist,” and “high risk.” The quality assessment was conducted by two researchers (KH L and YX L), and any disagreements were resolved through discussion with a third researcher (SW C).

### 2.5 Data analysis

In this study, R software (R4.5.1 Development Core Team, Vienna) was used to compare the post-intervention values of the intervention and control groups through meta-analysis to determine the effect of PMR on sleep quality. To assess the effect size, we used the standardized mean difference (SMD; Hedges g) and its 95% confidence interval (CI). A 95% confidence interval not passing through 0 indicated statistical significance; otherwise, it did not. Due to the clinical and methodological differences among the included studies, including differences in participant characteristics, chronic diseases, and intervention protocols, a random-effects model was used for the meta-analysis in this study. To improve the accuracy of the effect size, we used maximum likelihood estimation (REML) and applied Hartung–Knapp correction (25). Heterogeneity among different studies was assessed using *I*^2^: *I*^2^ < 25% indicated low heterogeneity, *I*^2^ between 26 and 50% indicated moderate heterogeneity, and *I*^2^ > 50% indicated high heterogeneity. When high heterogeneity (*I*^2^ > 50%) was observed, subgroup analyses were performed to explore the sources of heterogeneity. In this study, two subgroup analyses were conducted based on participants' origin (oncology and non-oncology patients) and age (Age < 55 and Age ≥55). The oncology subgroup was analyzed separately because it represented the largest and most clinically comparable patient population among the included studies, whereas the remaining studies involved diverse chronic conditions with insufficient numbers to support disease-specific subgroup analyses and were therefore combined into a non-oncology subgroup. When results suggested potential publication bias, the Trim-and-Fill method was further used to test the stability of the pooled effect size. Furthermore, a stepwise elimination method was employed for sensitivity analysis to assess the impact of individual studies on the overall effect. Finally, a meta-regression analysis was performed to explore whether the frequency of PMR was associated with sleep quality. Due to the limited number of included studies and the inconsistent reporting of other variables across studies, there was insufficient information to perform meta-regression analyses for these variables. Therefore, only the frequency of PMR was included in the meta-regression analysis in this study.

## 3 Results

### 3.1 Study selection

**Figure 1:**
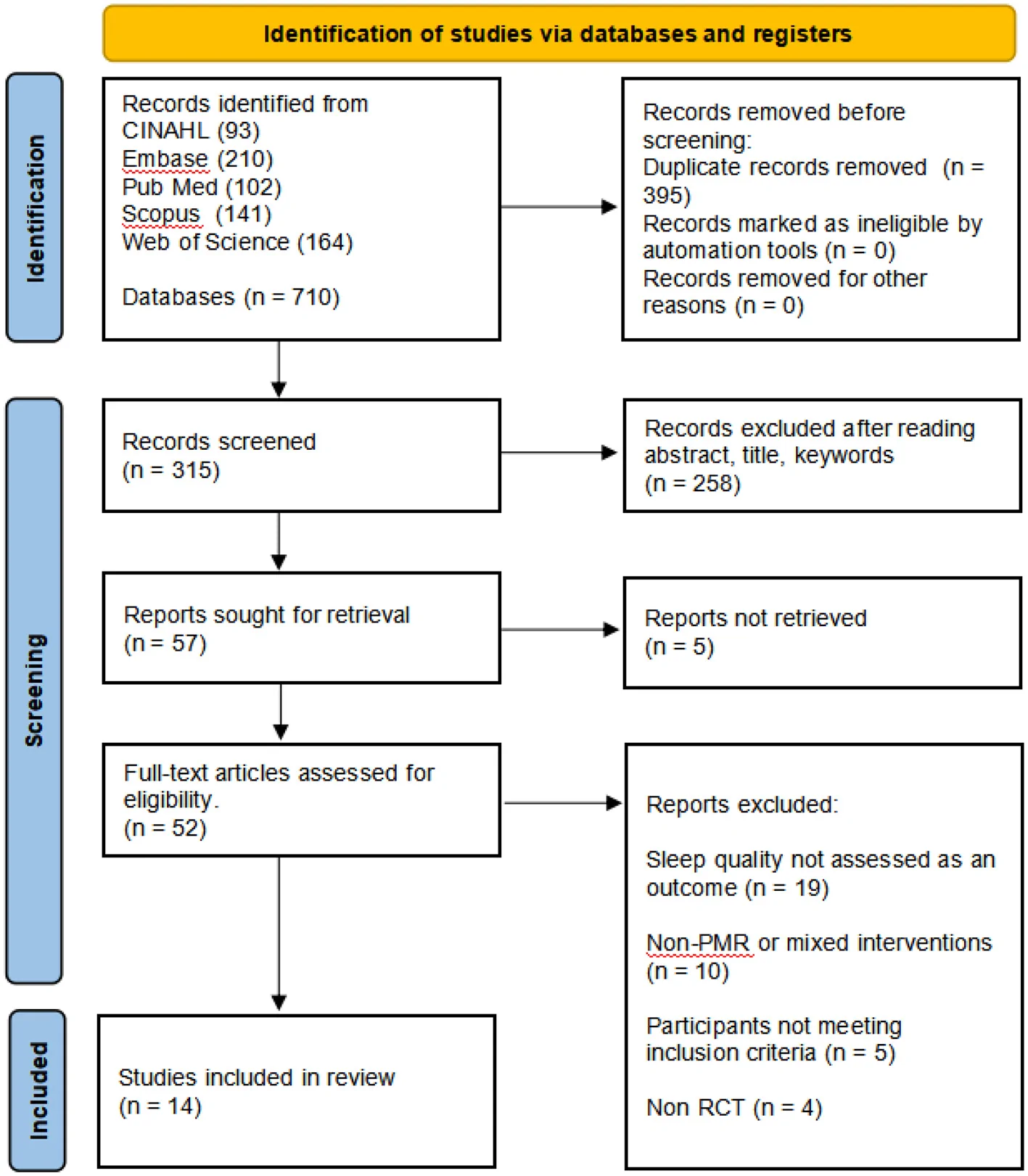
PRISMA flow diagram.

A systematic literature search was conducted in five databases: Cinahl, Embase, PubMed, Scopus, and Web of Science, yielding 710 records. After removing 395 duplicate records, 315 records remained. We initially excluded 258 records by reviewing titles, abstracts, and keywords. Of the 57 reports that passed the initial screening, 5 did not yield complete reports. The researchers reviewed the remaining 52 reports in full. 19 articles did not use the PSQI as an assessment of sleep quality; 10 studies did not use PMR or combine it with other interventions; 5 studies had participants under 18 years of age; and 4 studies were non-randomized controlled trials. A total of 14 studies met the inclusion criteria (18, 20–22, 26–35). See Figure 1 for details.

### 3.2 Assessing the quality of investigations

**Figure 2:**
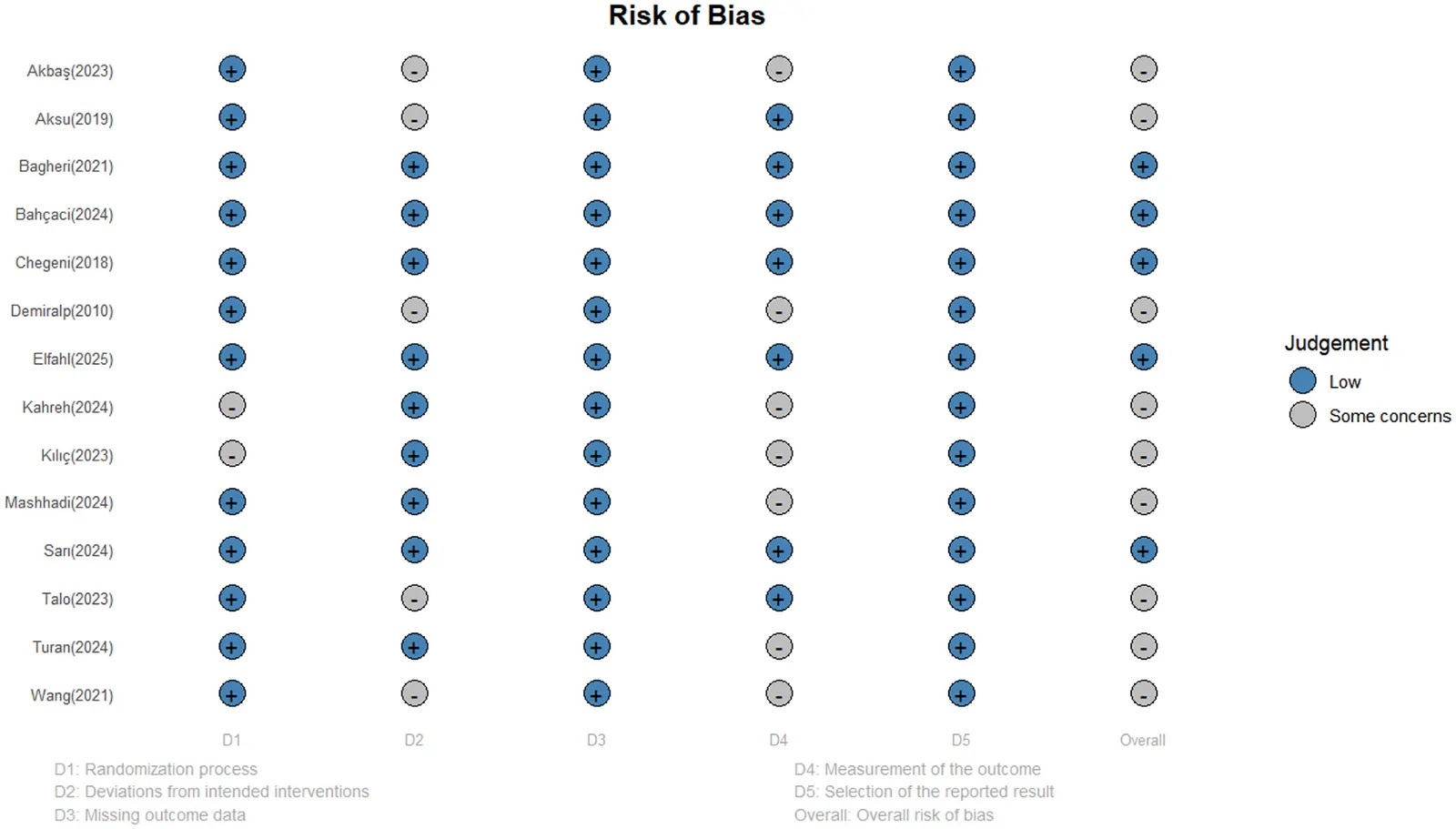
Assessing the Quality of Investigations.

As shown in Figure 2, of the 14 included articles, 5 were assessed as low-risk overall, and 9 as medium-risk. Two studies were rated as medium risk because the randomization procedure was not clearly described. Concerns related to participant blinding were identified in five studies, and assessor blinding concerns were identified in seven studies. All other domains were judged as low risk. Because the intervention methods differed across studies, blinding of participants and researchers was difficult to implement. However, the included studies generally showed acceptable methodological quality, with most studies falling into the low- or medium-risk categories.

### 3.3 Study characteristics

This systematic review included studies published between 2010 and 2025. This meta-analysis included 14 studies from four different countries (China (34), Turkey (20, 21, 28–32, 35), Iran (18, 22, 27, 33), and Egypt (26)). A total of 957 participants were included, of whom 447 were male and 510 were female (18, 20–22, 26–35). The participants included patients with cancer (20–22, 28, 29), coronary artery bypass graft (33), lung resection (35), restless legs syndrome (30), hemodialysis (26), rheumatoid arthritis (31), fractures (27), epilepsy (32), Chronic obstructive pulmonary disease (18) and Obstructive sleep apnea (34). All interventions were PMR, with controls including routine care (18, 21, 26, 28, 30, 33), no intervention (22, 27, 29, 31, 32, 34), light activity (20), and standard pulmonary rehabilitation therapy (35). Intervention durations ranged from 3 days to 12 weeks, with 4 (22, 32, 33) and 8 weeks (28–30) being common durations. The number of PMR sessions ranged from 3 to 112 (18, 20–22, 26–35). See Table 1 for details.

**Table 1:** Characteristics of studies included in the analysis.

References	Country	Sample size (E; C)		Female (%)	Age	Participant sources	Intervention (E; C)		Frequency and time	Out come
Elfahl & Elfeky (26)	Egypt	39	39	47%	E:49.4 ± 8.1C:49.53 ± 8.49	Hemodialysis	PRM	Routine care	3 session/weeks, 12 weeks	PSQI
Bahçaci et al. (20)	Turkey	26	26	100%	E:49.1 ± 8.8 C:44.7 ± 10	Breast cancer	PRM	Light activity	40 min/session, 3 session/weeks,6 weeks	PSQI
Kahreh et al. (22)	Iran	40	50	62%	45.95 ± 10.26	Cancer	PMR	No intervention	20 min/session, 2 session/day, 4 weeks	PSQI
Mashhadi-Naser et al. (27)	Iran	50	50	49%	E:53.78 ± 6.35 C:53.28 ± 6.66	Fractures	PRM	No intervention	30 min/session, 3 days	PSQI
Sari et al. (28)	Turkey	34	35	36%	E:54.26 ± 8.99 C: 52.74 ± 6.77	Cancer	PRM	Routine care	2 session/weeks, 8 weeks	PSQI
Turan et al. (29)	Turkey	37	37	41%	E:60.08 ± 11.99 C: 63.14 ± 7.79	Lung cancer	PRM	No intervention	30 min/session, 2 session/weeks, 8 weeks	PSQI
Akbaş & Sözbir (30)	Turkey	26	26	100%	E:28.03 ± 4.45 C: 26 ± 4.07	Restless legs syndrome	PRM	Routine care	3 session/weeks, 8 weeks	PSQI
Kiliç & Parlar Kiliç (31)	Turkey	35	37	78%	E: 46.3 ± 13.4 C : 56.6 ± 11.2	Rheumatoid arthritis	PRM	No intervention	2 session/day, 6 weeks	PSQI
Talo & Turan (32)	Turkey	35	35	47%	E: 41.97 ± 14.67 C: 42.69 ± 11.05	Epilepsy	PRM	No intervention	30 min/session,3session/weeks, 4weeks	PSQI
Bagheri et al. (33)	Iran	40	40	41%	E: 62.1 ± 6.3C:63.1 ± 7.6	Coronary artery bypass graft	PRM	Routine care	20 min/session,2 session/day,4 weeks	PSQI
Wang et al. (34)	China	38	38	22%	N/A	Obstructive sleep apnea	PRM	No intervention	40 min/session, 1 session/weeks, 12 weeks	PSQI
Aksu et al. (35)	Turkey	13	13	27%	E: 58.23 ± 13.5 C: 57.85 ± 6.95	Lung resection	PRM	Standard pulmonary rehabilitation therapy	20 min/session, 2 session/day, 7 days	PSQI
Chegeni et al. (18)	Iran	45	46	41%	E: 57.37 ± 12.8 C: 56.89 ± 14.6	Chronic obstructive pulmonary disease	PRM	Routine care	30 min/session, 10 session/weeks, 10 weeks	PSQI
Demiralp et al. (21)	Turkey	14	13	100%	N/A	Breast cancer	PRM	Routine care	8 session/78 days	PSQI

E, experimental group; C, control group; PMR, progressive muscle relaxation; PSQI, Pittsburgh Sleep Quality Index.

### 3.4 Meta-analysis

#### 3.4.1 Effects of progressive muscle relaxation on sleep quality

**Figure 3:**
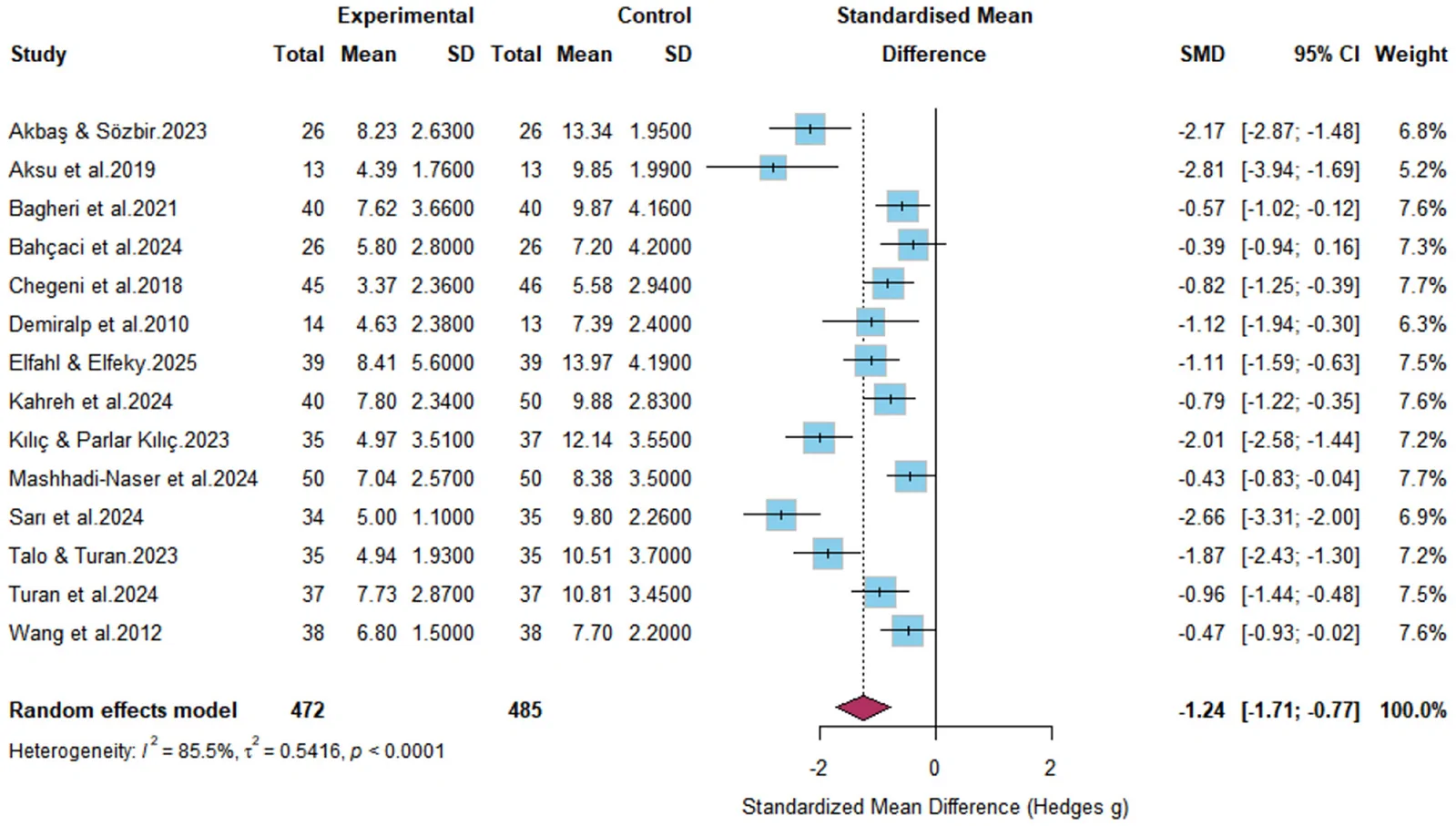
Forest diagram of the effects of PMR on sleep quality.

The meta-analysis was based on 14 studies, including 472 participants in the intervention group and 485 participants in the control group. The pooled effect size was statistically significant (Hedges *g* = −1.24, 95% CI: −1.71–−0.77), and the 95% confidence interval did not cross 0. This finding suggests that PMR significantly improved sleep quality. The *I*^2^ value was 85.5%, indicating substantial heterogeneity across studies. Details are provided in Figure 3.

#### 3.4.2 Subgroup analysis

Because substantial heterogeneity was observed (*I*^2^ = 85.5%), subgroup analyses were carried out to identify potential sources of variation. Participants were stratified by disease status (cancer patients vs. non-cancer patients) and age group (Age < 55 vs. Age ≥55) for subgroup analyses.

**Figure 4:**
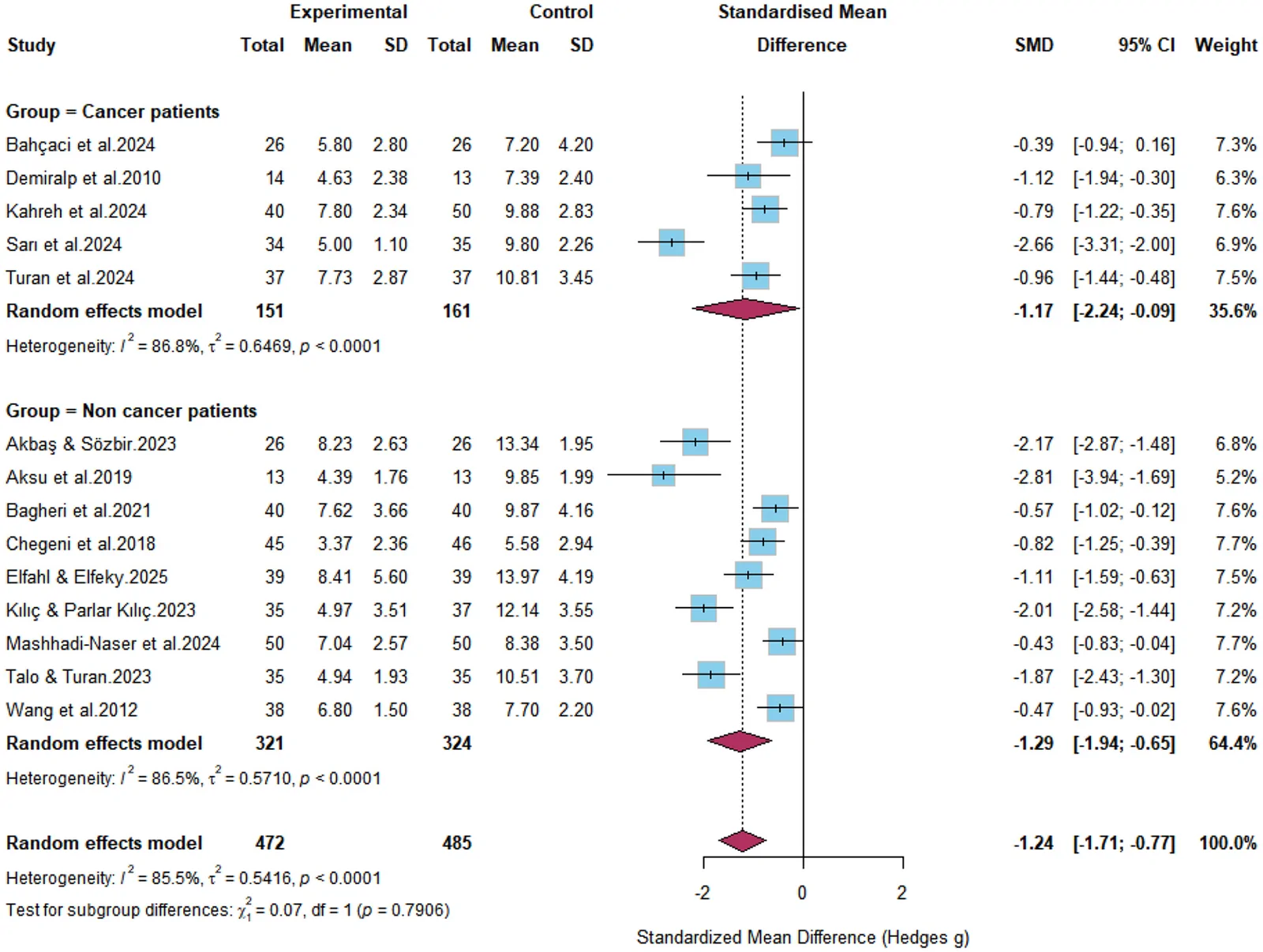
Forest diagram of subgroup analysis of participant sources.

In the subgroup analysis based on participants' origin (cancer patients and non-cancer patients), 5 studies included cancer patients, and 9 studies included patients with other diseases. PMR was associated with improved sleep quality in both cancer and non-cancer patients; however, these subgroup analyses did not account for the observed heterogeneity. For cancer patients, the pooled effect size was statistically significant (Hedges *g* = −1.17, 95% CI −2.24–−0.09), with substantial heterogeneity (*I*^2^ = 86.8%). A significant pooled effect was also observed among non-cancer patients (Hedges *g* = −1.29, 95% CI −1.94–−0.65), accompanied by high heterogeneity (*I*^2^ = 86.5%). See Figure 4 for details.

**Figure 5:**
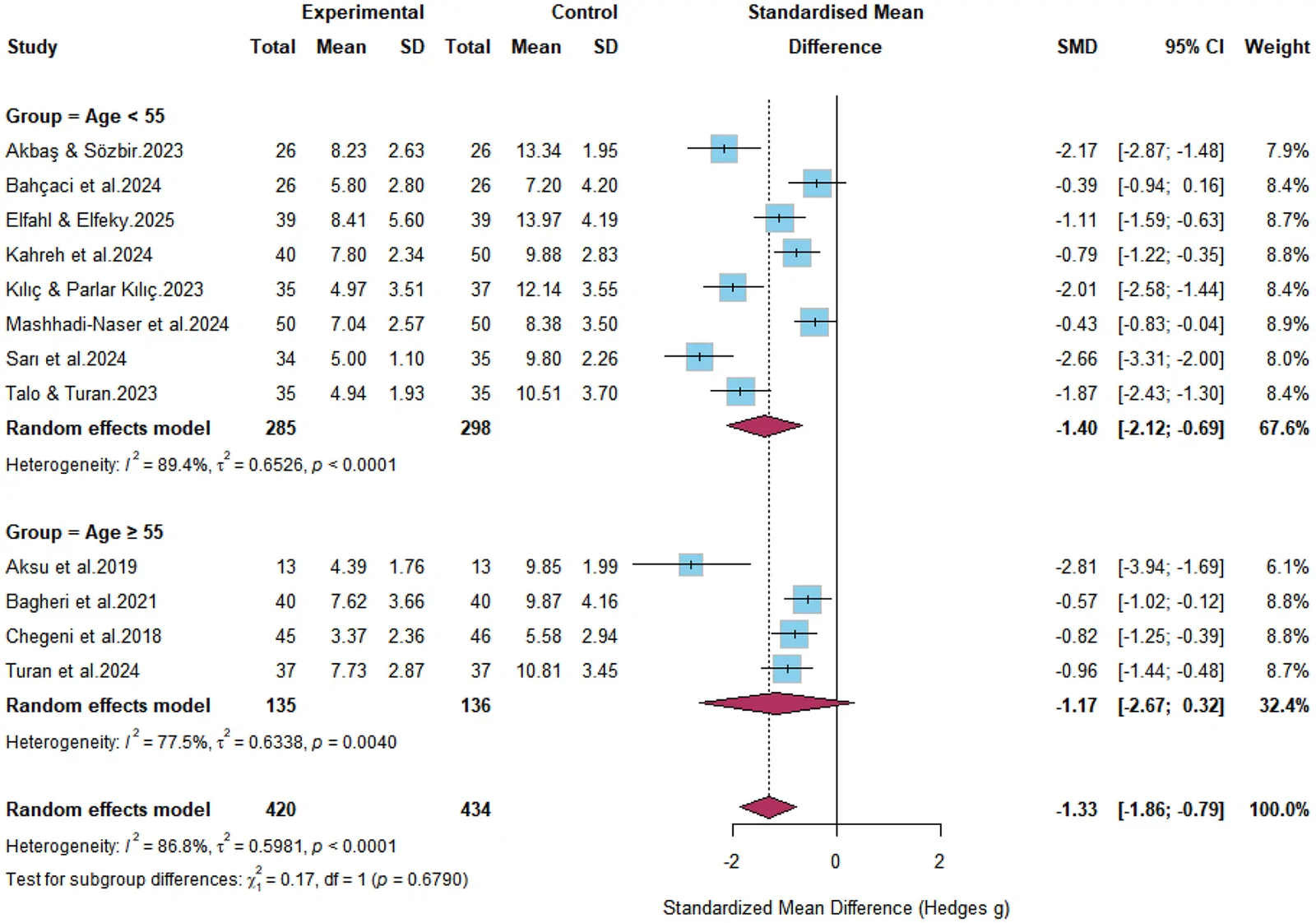
Forest diagram of subgroup analysis of age.

Subgroup analyses based on participants' age (Age < 55 and Age ≥55) excluded two studies due to failure to report specific ages; eight studies had participants aged < 55 years; and four studies had participants aged ≥55 years. The results indicate that PMR improves sleep quality in adults < 55 years, but has no effect on older adults aged ≥55 years, with high heterogeneity in both Age < 55 and Age ≥55 subgroups. The effect size for adults aged < 55 (Hedges *g* = −1.4, 95% CI −2.12, −0.69) did not include 0 within the 95% confidence interval, *I*^2^ = 89.4%. The effect size for older adults aged ≥55 (Hedges *g* = −1.17, 95% CI −2.67, 0.32) also included 0 within the 95% confidence interval, *I*^2^ = 77.5%. However, the test for subgroup differences was not statistically significant (χ^2^ = 0.17, df = 1, *p* = 0.679), indicating that the intervention effect did not differ significantly between the two age groups. See Figure 5 for details.

### 3.5 Regression results of progressive muscle relaxation numbers on sleep quality

**Figure 6:**
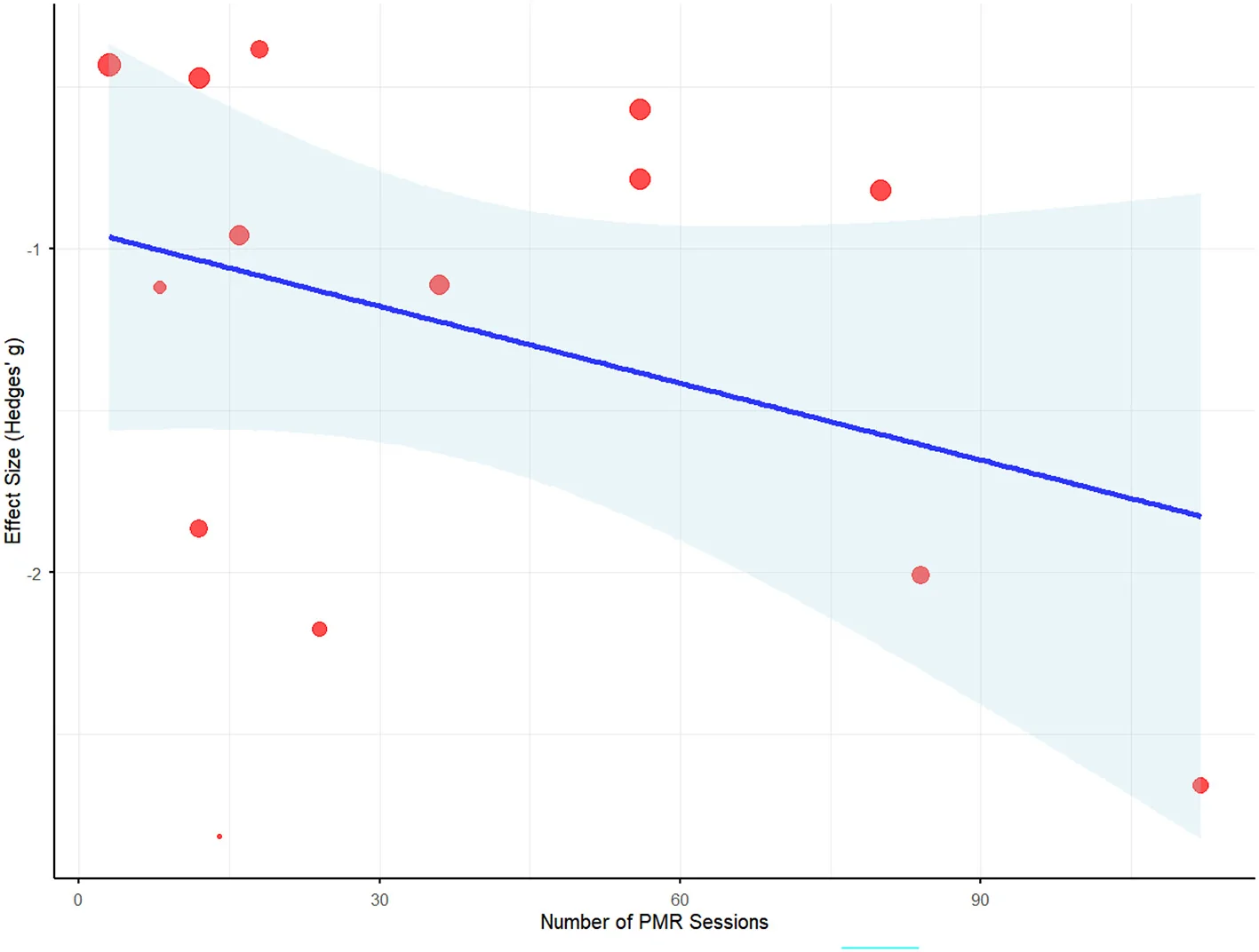
Meta-regression analysis of the effect of PMR on sleep quality.

Meta-regression was used to examine whether the number of PMR sessions was related to sleep quality. No significant association was found between the number of PMR sessions and the sleep quality effect size (Hedges' g) (β = −0.0079, 95% CI: −0.0203–0.0045, *p* = 0.2106). This indicates that the available evidence does not support a significant effect of session number on sleep quality. See Figure 6 for details.

### 3.6 Sensitivity analysis

**Figure 7:**
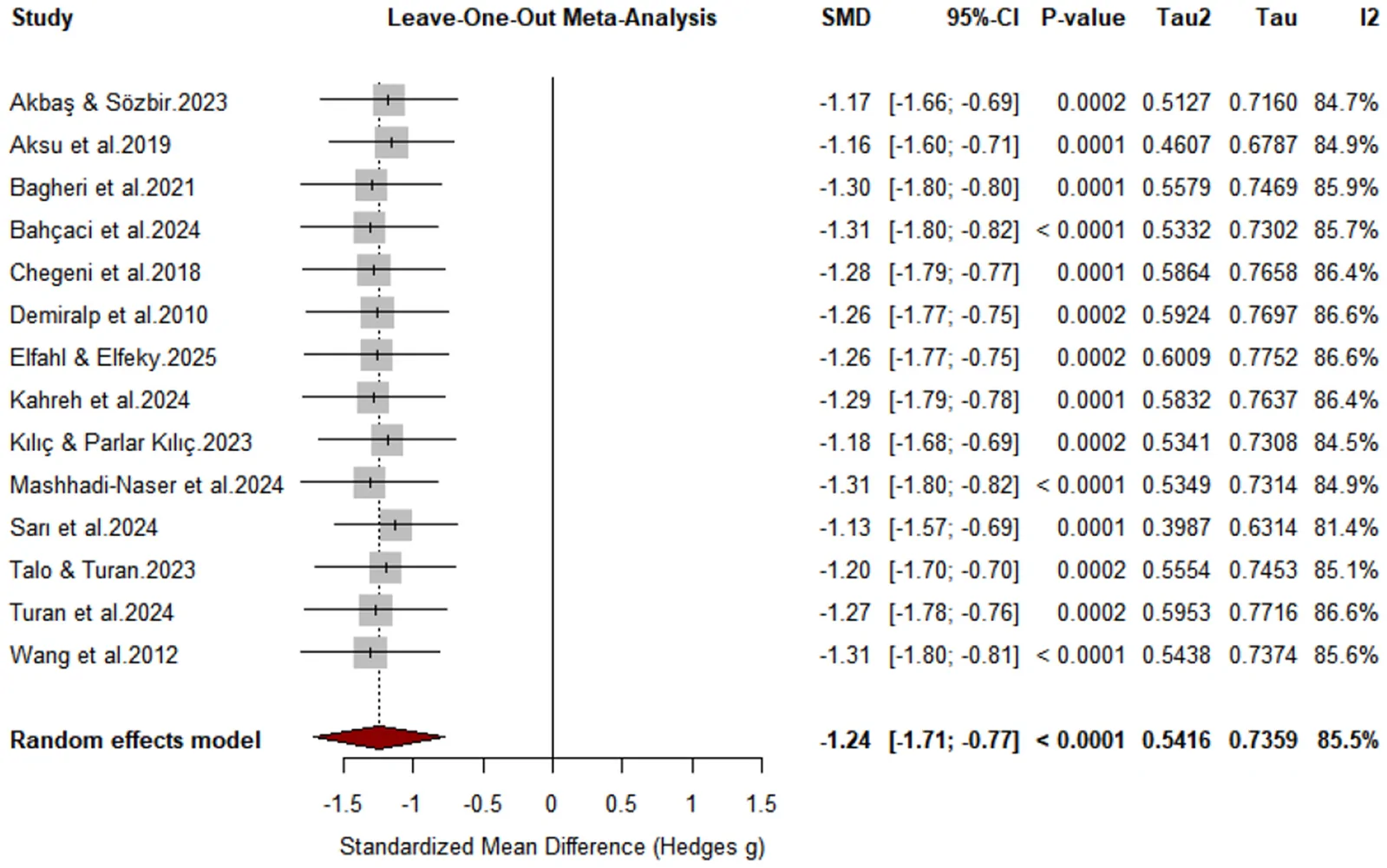
Leave-one-out sensitivity analyses.

Sensitivity analysis was conducted using a leave-one-out approach. The pooled effect size remained stable after each study was removed in turn. The pooled Hedges' g ranged from −1.13–−1.31. None of the 95% confidence intervals contained 0, and the direction of the effect sizes remained unchanged. The *I*^2^ ranged from 81.4 to 86.6, and Tau^2^ (0.4–0.6) and Tau (0.63–0.77) were relatively stable, indicating that no single study significantly affected heterogeneity. These results collectively demonstrate that no single study in this meta-analysis altered the overall outcome, indicating high stability of the results. See Figure 7 for details.

### 3.7 Publication bias

**Figure 8:**
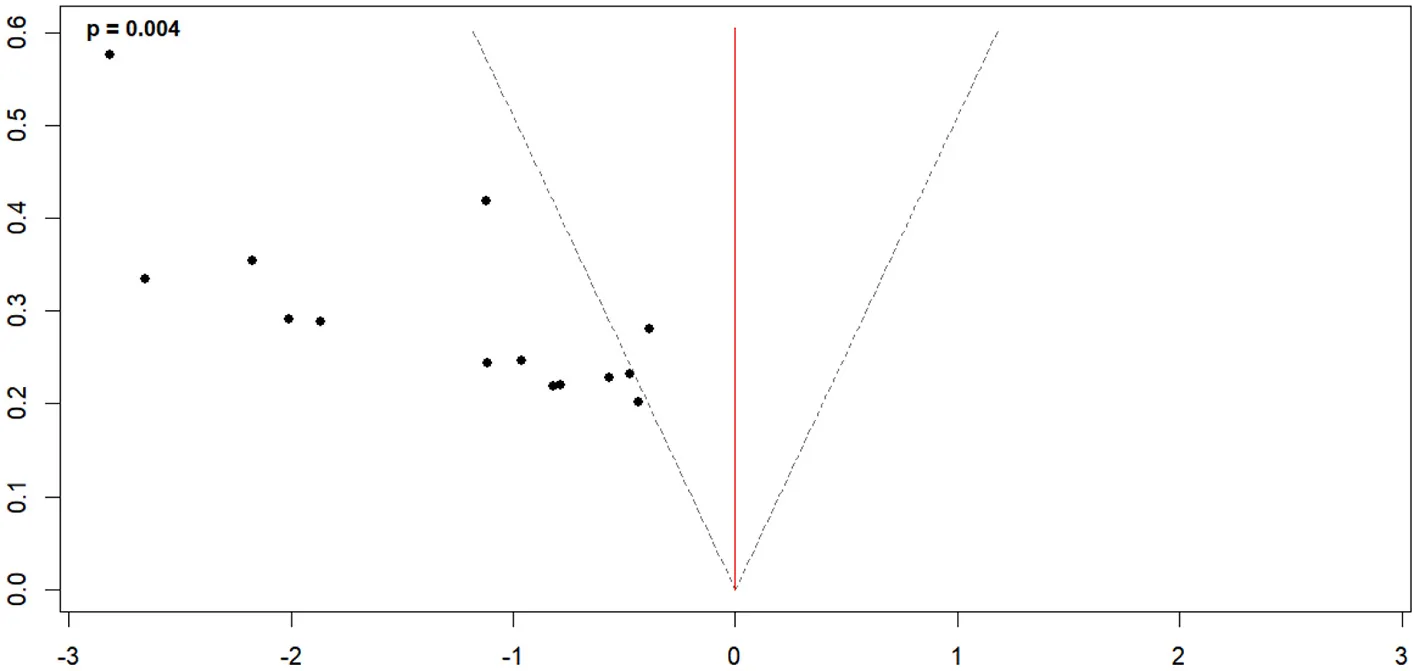
The funnel plot for publication bias and Egger test.

Publication bias was examined using funnel plots and Egger's test. As shown in Figure 8, the funnel plot appeared asymmetric and suggested possible bias. Egger's test yielded a *p*-value of 0.004, which was below 0.05, indicating possible publication bias. We corrected for this bias using the Trim-and-Fill method. After adjustment, *K* = 17, with three studies added, suggesting that three studies may have been missing. The adjusted effect size (Hedges *g* =-0.92, 95% CI −1.49, −0.36) remained statistically significant, and the effect direction remained unchanged, indicating that the overall results of this study were relatively stable.

## 4 Discussion

This study synthesized available evidence on the effects of PMR on adult sleep quality. The pooled results indicated that PMR significantly improved sleep quality in adults. Further subgroup analyses found that sleep quality was significantly improved through PMR in both cancer patients and non-cancer patients. From an age perspective, this intervention significantly improved sleep quality in adults aged < 55 years, but had no significant effect on sleep quality in older adults aged ≥55 years. Meta-regression analysis showed no significant association between exercise frequency and sleep quality. In addition, significant heterogeneity was observed among the included studies.

Regarding the reasons why PMR can improve sleep quality, it may be because people's sleep and wakefulness are usually affected by sleep pressure and arousal threshold (36). PMR, combined with regular breathing and the contraction and relaxation of muscles throughout the body, may relieve sympathetic nerve excitation and reduce cortisol (14). Reduced sympathetic nerve activity and cortisol levels may help release sleep pressure and lower the arousal threshold, thereby contributing to improvements in sleep quality (37, 38). Another reason may be that the improvement of sleep quality by PMR is due to the mediating effect of anxiety and depression. All participants in this study had different degrees of illness, which can lead to depression and anxiety (39). However, there is a mutual influence between anxiety and depression and sleep (40). Because PMR may reduce anxiety and depression, these psychological changes may partly explain its effect on sleep quality (15).

The subgroup findings suggested beneficial effects of PMR in both cancer and non-cancer patients. Although a larger pooled effect size was observed in the non-cancer subgroup, this finding should be interpreted with caution because the non-cancer subgroup included participants with clinically heterogeneous conditions. One study showed that cancer patients had more difficulty falling asleep and staying asleep than other patients (41). Cancer and cancer-related treatments can lead to an increase in serotonin levels, which in turn leads to an increase in norepinephrine and sympathetic nerve activity (42, 43). However, norepinephrine disturbances and sympathetic nerve imbalances can severely affect sleep quality (44, 45). These factors may reduce the sleep-related benefits of PMR in cancer patients. In age-related subgroups, the age-based analysis indicated a significant effect in adults younger than 55 years, while no significant effect was observed in older adults. However, the test for subgroup differences was not statistically significant, indicating that the intervention effect did not differ significantly between the two age groups. Therefore, these findings should be interpreted with caution. As people age, their need for sleep does not change (46). The amplitude of slow-wave brain activity decreases, and multiple chronic diseases coexist (36). This makes it harder for older adults to fall asleep and easier to wake up. The improvement in sleep quality from PMR may diminish with age, although this finding should be interpreted with caution. Unfortunately, despite our subgroup analysis, we have not yet identified the source of this heterogeneity. This indicates that participant type and age alone are insufficient to fully explain the differences among studies. The sources of heterogeneity may be influenced by variations in intervention protocols, disease characteristics, and intervention duration. Therefore, although the overall results consistently support the improvement of subjective sleep quality by PMR, the pooled findings should still be interpreted with caution. Moreover, the substantial heterogeneity among the included studies may limit the generalizability of the findings to specific clinical populations and intervention settings.

Meta-regression did not show a significant link between the number of PMR sessions and sleep quality. This suggests that the frequency is not a key factor in improving sleep quality. The study found that performing PMR once a day for three consecutive days and twice a day for eight consecutive weeks improved sleep quality, which confirms this view (27, 28).

### 4.1 Limitations and future directions

This meta-analysis has several limitations. First, the heterogeneity of sleep quality in this study was high. Although subgroup analyses were conducted, this heterogeneity could not be fully resolved. The high heterogeneity may stem from factors such as participants' disease types and the duration of intervention. In addition, the non-oncology subgroup included participants with various chronic conditions. Owing to the limited number of studies available for each condition, disease-specific subgroup analyses were not feasible, which may have introduced additional clinical heterogeneity. Therefore, the findings of the non-oncology subgroup should be interpreted with caution. Second, publication bias testing revealed some publication bias in this study. While the Trim-and-Fill method did not change the direction of the effect size, it did reduce it; therefore, the results should be interpreted with caution. Third, this study used the PSQI, which may introduce subjectivity; the results should be interpreted with caution.

Sleep is a complex physiological process. Future research on the effects of PMR on sleep could consider using a method that simultaneously measures subjective and objective factors. Furthermore, sleep is a long-term process; future research should include follow-up after intervention to verify the long-term effectiveness of PMR in improving sleep. Additionally, given individual variability, future studies should also investigate whether different groups will show different results. For example, differences in gender, disease, and whether relaxation is group-based or individual.

## 5 Conclusion

This systematic review and meta-analysis found that PMR can improve subjective sleep quality in adults, showing beneficial effects in both cancer and non-cancer patients. However, the effects of progressive muscle relaxation on subjective sleep quality in adults aged ≥55 years remain uncertain. These findings support the use of progressive muscle relaxation as a non-pharmacological intervention for improving subjective sleep quality and may help inform future clinical practice and research.

## Data Availability

The original contributions presented in the study are included in the article/Supplementary material, further inquiries can be directed to the corresponding author.
