# Supplementary material for: The effects of progressive muscle relaxation on sleep quality in adults: a systematic review and meta-analysis of randomized controlled trials
Source: Front Public Health. 2026 Aug 6;14:1906525. doi: 10.3389/fpubh.2026.1906525 (PMC13489916; doi:10.3389/fpubh.2026.1906525)
Supplement: Supplementary file 1 [file Table_1.DOCX]

**Appendix 1：search strategy**

| **Database** | **Retrieval strategy** |
| --- | --- |
| **CINAHL** | ((MH "Muscle Relaxation")  OR TI ("progressive muscle relaxation" OR "muscle relaxation therapy" OR "Jacobson relaxation" OR "Jacobson technique" OR PMR)  OR AB ("progressive muscle relaxation" OR "muscle relaxation therapy" OR "Jacobson relaxation" OR "Jacobson technique" OR PMR))  AND  ((MH "Sleep")  OR (MH "Sleep Wake Disorders")  OR TI (sleep OR "sleep quality" OR insomnia OR "sleep disturbance" OR "sleep disturbances" OR "sleep disorder" OR "sleep disorders" OR "sleep problem" OR "sleep problems")  OR AB (sleep OR "sleep quality" OR insomnia OR "sleep disturbance" OR "sleep disturbances" OR "sleep disorder" OR "sleep disorders" OR "sleep problem" OR "sleep problems"))  AND  ((MH "Randomized Controlled Trials")  OR PT Clinical Trial  OR TI (randomized OR randomised OR randomly OR trial)  OR AB (randomized OR randomised OR randomly OR trial)) |
| **Embase** | ('exp muscle relaxation'/OR 'progressive muscle relaxation':ti,ab,kw OR 'muscle relaxation therapy':ti,ab,kw OR 'jacobson relaxation':ti,ab,kw OR 'jacobson technique':ti,ab,kw OR PMR:ti,ab,kw)  AND  ('exp sleep'/OR 'exp sleep disorder'/OR sleep:ti,ab,kw OR 'sleep quality':ti,ab,kw OR insomnia:ti,ab,kw OR 'sleep disturbance':ti,ab,kw OR 'sleep disturbances':ti,ab,kw OR 'sleep disorder':ti,ab,kw OR 'sleep disorders':ti,ab,kw OR 'sleep problem':ti,ab,kw OR 'sleep problems':ti,ab,kw)  AND  ('exp randomized controlled trial'/ OR 'controlled clinical trial':ti,ab,kw OR randomized:ti,ab,kw OR randomised:ti,ab,kw OR randomly:ti,ab,kw OR trial:ti,ab,kw) |
| **PubMed** | ("Progressive Muscle Relaxation"[Mesh] OR "progressive muscle relaxation"[Title/Abstract]OR "muscle relaxation therapy"[Title/Abstract] OR"Jacobson relaxation"[Title/Abstract] OR "Jacobson technique"[Title/Abstract] OR PMR[Title/Abstract])  AND  ("Sleep"[Mesh] OR "Sleep Wake Disorders"[Mesh] OR sleep[Title/Abstract] OR "sleep quality"[Title/Abstract] OR insomnia[Title/Abstract]OR "sleep disturbance"[Title/Abstract] OR "sleep disturbances"[Title/Abstract] OR "sleep disorder"[Title/Abstract] OR "sleep disorders"[Title/Abstract] OR "sleep problem"[Title/Abstract] OR "sleep problems"[Title/Abstract])  AND  (Randomized Controlled Trial[Publication Type] OR Controlled Clinical Trial[Publication Type] OR randomized[Title/Abstract] OR randomised[Title/Abstract] OR randomly[Title/Abstract] OR trial[Title/Abstract]) |
| **Scopus** | TITLE-ABS-KEY (("progressive muscle relaxation" OR "muscle relaxation therapy" OR "Jacobson relaxation" OR "Jacobson technique" OR PMR)  AND  (sleep OR "sleep quality" OR insomnia OR "sleep disturbance" OR "sleep disturbances" OR "sleep disorder" OR "sleep disorders" OR "sleep problem" OR "sleep problems")  AND  ("randomized controlled trial" OR "controlled clinical trial" OR randomized OR randomised OR randomly OR trial)) |
| **Web of Science** | TS=(("progressive muscle relaxation"OR "muscle relaxation therapy"OR "Jacobson relaxation"OR "Jacobson technique"OR PMR)  AND  (sleep OR "sleep quality"OR insomnia OR "sleep disturbance"OR "sleep disturbances"OR "sleep disorder"OR "sleep disorders"OR "sleep problem"OR "sleep problems")  AND  ("randomized controlled trial"OR "controlled clinical trial"OR randomized OR randomised OR randomly OR trial)) |
